# Diversity and Dosage Determine the Efficacy of the Probiotic SYN‐53 in Allergic Rhinoconjunctivitis: A Randomized, Double‐Blind, Placebo‐Controlled Trial

**DOI:** 10.1111/all.70045

**Published:** 2025-09-10

**Authors:** Karl‐Christian Bergmann, Torsten Zuberbier

**Affiliations:** ^1^ Institute of Allergology, Charité Universitätsmedizin, Berlin Corporate Member of Freie Universität Berlin and Humboldt Universität Zu Berlin Berlin Germany; ^2^ Fraunhofer Institute for Translational Medicine and Pharmacology ITMP, Immunology and Allergology Berlin Germany

**Keywords:** allergen exposure chamber, allergic rhinoconjunctivitis, bacterial diversity, microbiome, multi‐strain probiotic

## Abstract

SYN‐53, a multi‐strain probiotic food supplement, was recently shown to significantly alleviate allergic rhinoconjunctivitis (ARC) and its symptoms. The diversity and dosage of bacterial strains administered via SYN‐53 have been proposed as key drivers of its efficacy. The aim of this study was to assess the role of bacterial diversity and dosage by comparing SYN‐53 to a low dose variant (SYN‐53‐LD), a low diversity variant (SYN‐4), and a placebo in the management of ARC. This double‐blind, parallel‐group, placebo‐controlled clinical trial included subjects with moderate‐to‐severe grass pollen allergy. Following baseline exposure in an allergen exposure chamber (AEC), 166 subjects were randomized to undergo three weekly 3‐day intake cycles of SYN‐53, SYN‐53‐LD, SYN‐4, or placebo, followed by a final allergen exposure. During AEC exposure, symptoms were continuously measured via assessment of the Total Symptom Score (TSS). SYN‐53 was significantly superior in reducing TSS_MAX_ compared with its low dose variant SYN‐53‐LD (∆TSS_MAX_ [Mean ± SE]: −5.19 ± 0.80 vs. −2.27 ± 0.65; *p* = 0.0372), its low diversity variant SYN‐4 (−3.41 ± 0.52; *p* = 0.0482), and placebo (−2.82 ± 0.78; *p* = 0.0329). No significant differences to placebo were seen for either SYN‐53‐LD (*p* = 0.7377) or SYN‐4 (*p* = 0.5152). SYN‐53 and its variants were well tolerated, and adverse events were not different from placebo. Our findings reaffirm the efficacy of SYN‐53 in the management of ARC and demonstrate that the effectiveness of this multi‐strain probiotic is intricately linked to the diversity of bacterial strains and dosage administered.

## Graphical abstract

The probiotic food supplement SYN‐53 has previously shown efficacy in managing allergic rhinoconjunctivitis (ARC), with its high bacterial diversity and dosage hypothesized as key drivers. This study on 166 subjects aimed to confirm this hypothesis by comparing the effects of SYN‐53, a low dose variant (SYN‐53‐LD), a low diversity variant (SYN‐4), and a placebo on ARC in an allergen exposure chamber. SYN‐53 demonstrated significant superiority over all variants and placebo in reducing symptoms of ARC, confirming that its effectiveness is intricately linked to high bacterial diversity and dosage. *, *p* < 0.05; AEC, allergen exposure chamber; ARC, allergic rhinoconjunctivitis; CFU, colony forming units; LD, low dose; TSS_MAX_ , maximal Total Symptom Score.

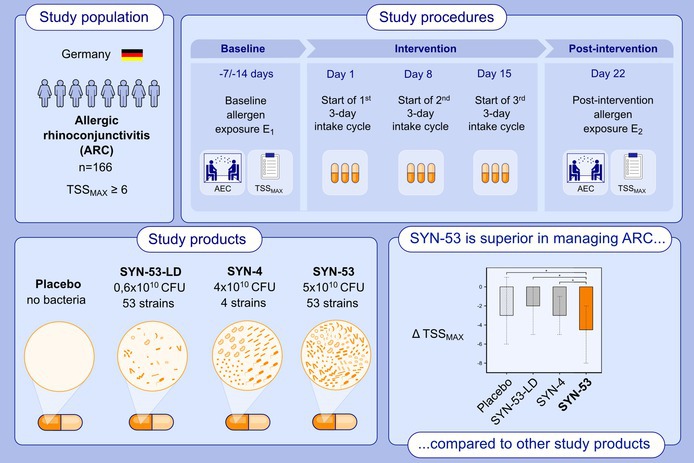

## 1 Introduction

One of the main functions of the immune system is to distinguish between potentially harmful and harmless stimuli and balance its responses accordingly. Disruption of this balance, particularly the loss of immune tolerance, can lead to the development of autoimmune diseases and allergic conditions [1, 2, 3]. The gut microbiome is now recognized as a key modulator of immune function, influencing epithelial barrier integrity and immune homeostasis through complex bidirectional host–microbe interactions [4, 5]. Consequently, the gut microbiome has emerged as a critical factor in the pathogenesis of allergies over the past decades, with growing evidence linking microbial composition and function to allergy susceptibility [1, 6, 7, 8].

One particularly prevalent chronic allergic condition is allergic rhinitis (AR), affecting approximately 400 million people worldwide [9, 10]. AR is caused by immunoglobulin E (IgE)‐mediated reactions to inhaled allergens, such as plant‐derived pollen, which commonly leads to allergic symptoms, such as rhinorrhea, sneezing, nasal itching, and congestion. Allergic rhinoconjunctivitis (ARC) refers to the co‐occurrence of AR and ocular symptoms, such as itching, burning, redness, or teary eyes [11]. ARC has the highest prevalence rates in high‐income countries (up to 40%) causing steadily increasing economic costs [10, 12]. Interestingly, it was recently shown that the microbiome of patients with ARC is associated with distinctive dysbiotic patterns, which include, for example, a higher abundance of Bacteroidetes species and a reduction in the levels of Firmicutes and Clostridiales when compared to those of healthy controls [13, 14, 15, 16]. Therefore, modulating the microbiome to restore its natural balance and bacterial composition appears to be a promising approach to the management of ARC. Two common methods to modulate the gut microbiome, probiotics, and fecal microbiota transplantation (FMT), have demonstrated the ability to promote immune balance [17]. Probiotics in food or food supplements typically deliver a defined set of selected, viable bacterial strains, whereas FMT transfers entire, often uncharacterized microbial communities from the feces of one or more healthy donors [17].

Several studies have demonstrated that such modulations of the gut microbiome can improve symptoms of allergic diseases [18, 19, 20, 21, 22, 23, 24]. In particular, consumption of probiotics for the management of ARC has been widely reported by clinical trials and recent meta‐analyses, which indicate improvements in symptoms and quality of life, though with high heterogeneity of results [21, 22, 25, 26, 27]. These studies often use a small number of specific bacterial strains (1–3), and differences in dosage and administration are notable, with the efficacy of multi‐strain preparations superior to single‐strain probiotics [23]. Even though some initial studies applied FMT in the context of allergic conditions [18, 19, 20], such as atopic dermatitis or food allergies, there are many reservations due to the low level of standardization and scalability, as well as the risk of transmission of infectious agents [28, 29], limiting its application to the treatment of more severe conditions, such as recurrent *Clostridioides difficile* infections, metabolic syndrome, and ulcerative colitis [30, 31, 32, 33].

Building on this, SYN‐53 was designed as a nutritional supplement containing 53 viable bacterial strains, delivered at a daily dose of 1.5 × 10^11^ colony forming units (CFU). SYN‐53 was recently investigated in a double‐blind, randomized, placebo‐controlled clinical study in the management of ARC [34]. Over the course of three bi‐weekly 3‐day intake cycles, SYN‐53 was shown to significantly alleviate ARC and its symptoms. It has been hypothesized that the efficacy of SYN‐53 is largely attributable to the diversity and dosage of bacterial strains administered. This follow‐up double‐blind, randomized, placebo‐controlled clinical study aimed to confirm these previous findings by comparing the efficacy and safety of the nutritional supplement SYN‐53 with a low dose variant (SYN‐53‐LD), a low diversity variant (SYN‐4), and a placebo under the standardized conditions of an allergen exposure chamber (AEC).

## 2 Methods

### 2.1 Study Design

A double‐blind, placebo‐controlled, monocentric, randomized, parallel‐group clinical study was carried out in the GA^2^LEN allergen exposure chamber located at the European Centre for Allergy Research Foundation (ECARF) in Berlin (Germany) from September to December 2022. The autumn/winter months were chosen to avoid any confounding influences of the natural grass pollen season.

**FIGURE 1:**
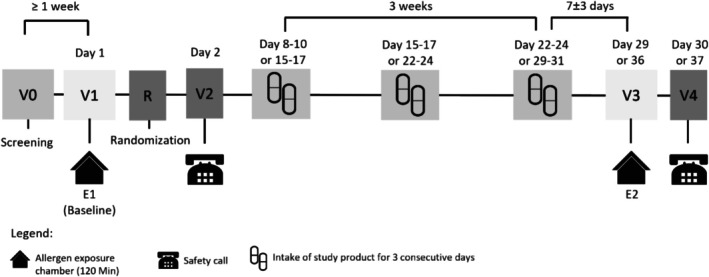
Study timeline. The study was started with a screening visit (V0), followed by baseline exposure (E1) in the allergen exposure chamber (AEC) the next day (V1) and a safety call on day two (V2). Upon randomization (R) to the study group, subjects underwent three consecutive weeks of intake cycles with the study product. For scheduling reasons with regard to the post‐intervention exposure (E2) at visit three (V3), subjects started the intake of the study product 7 or 14 days after E1 for a total of 3 weeks. Irrespective of intake start, the post‐intervention exposure E2 occurred at V3 1 week (±3 days) after the start of the last intake cycle. The study was ended with a final safety call (V4) 1 day after the post‐intervention exposure.

The timeline of the study is shown in Figure 1. The study consisted of a screening visit (V_0_) followed by a baseline exposure (Exposure 1; E_1_) in the AEC to identify subjects with moderate‐to‐severe symptoms (see 2.2 Participants). Upon randomization into either one of the four study groups (SYN‐53, SYN‐53‐LD, SYN‐4, or placebo; see 2.3), each subject underwent three consecutive weekly intake cycles. Each intake cycle consisted of 3 days of investigational product intake (three capsules per day at mealtime), followed by a four‐day break. Finally, a second (post‐intervention) exposure (E_2_) in the AEC was held 1 week (±3 days) after the start of the third intake cycle. Safety phone calls (V_2_ and V_4_) were conducted 1 day after each allergen exposure to inquire about subjects' well‐being after allergen exposure, the use of safety medication, and the tolerability of the study product.

All allergen exposures took place at the Global Allergy and Asthma European Network (GA^2^LEN) AEC located at the European Centre for Allergy Research Foundation (ECARF) in Berlin (Germany). The GA^2^LEN AEC consists of two connected mobile containers accommodating a test chamber for up to nine participants plus a study nurse, a changing room, and an observation office. A detailed description of the chamber can be found in Zuberbier et al. Participants were exposed to a standardized grass pollen ( *Phleum pratense* ) preparation (Stallergenes Greer, Lenoir, USA) at a rate of 4.000 pollen/m^3^ for 120 min at room temperature (20°C) and 55% relative humidity.

The study protocol has been approved by the ethics committee of the Ethics Commission of Charité Universitätsmedizin Berlin (E1/128/22) and was conducted in accordance with the Declaration of Helsinki. The trial is registered at the ISRCTN clinical trial registry (ISRCTN15622522). All subjects provided informed consent.

### 2.2 Participants

The study population consisted of subjects aged between 18 and 65 years with clinically relevant sensitization to grass pollen, a history of allergic symptoms for at least 2 years, a positive skin prick test to grass pollen (wheal diameter ≥ 3 mm) and moderate‐to‐severe response to grass pollen during exposure in the AEC (TSS_MAX_ ≥ 6) at E_1_.

Exclusion criteria included acute infections, current cancer diagnosis or cancer in the 5 years prior to the study, autoimmune diseases, gastrointestinal diseases causing diminished metabolic processing of orally taken substances, and severe types of one of the following chronic diseases: neurological diseases, metabolic diseases, severe asthma, severe pulmonary obstruction, innate anomalies of the heart, gastrointestinal tract, or lung. Furthermore, subjects suffering from psychological diseases (e.g., depression) or eating disorders (e.g., bulimia) in the 2 years prior to the study, alcohol or drug addiction, allergies to any of the ingredients of the preparations tested, and counter indications against adrenaline or any other rescue medication (especially cetirizine), were excluded. In addition, subjects with a forced expiratory volume (FEV) < 70% prior to allergen exposure in the AEC, pregnant or breastfeeding women, heavy smokers (≥ 20 cigarettes/day), subjects who had undergone specific immune therapy in the 5 years prior to the study, and subjects who had participated in clinical trials in the 3 months prior to the screening procedure were not admitted.

The following concomitant medications and preparations had to be paused prior to E_1_ and were not allowed during the study: decongestant nasal drops (3 days prior E_1_), antihistamines (5 days prior E_1_), anti‐allergic eye drops and nasal sprays (1 week prior E_1_), topical steroids (2 weeks prior E_1_), systemic corticosteroids (3 weeks prior E_1_), probiotics (4 weeks prior E_1_), and antibiotics (4 weeks prior E_1_).

### 2.3 Study Products and Rescue Medication

SYN‐53, SYN‐53‐LD, and SYN‐4 are three variants of the nutritional supplement SYN‐53. SYN‐53 contains 53 distinct lyophilized bacterial strains at a dosage of 5 × 10^10^ CFU/capsule. SYN‐53‐LD contains the same 53 bacterial strains, but at a considerably lower dosage of only 0.6 × 10^10^ CFU/capsule (one log‐level below SYN‐53). SYN‐4, in contrast, is dosed similarly to SYN‐53 (4 × 10^10^ CFU/capsule) but contains only four bacterial strains of SYN‐53. The bacterial composition of each variant is listed in Table S1. All bacterial species contained in either SYN‐53 variant are commonly found in food and have been granted qualified presumption of safety (QPS) status by the European Food Safety Authority (EFSA). All SYN‐53 variants contain maize starch as a filling agent and magnesium stearate and silicon dioxide as anti‐caking agents. All SYN‐53 variants were encapsulated in small size 3 hydroxypropyl methyl cellulose capsules for oral administration to enhance stability, ease of use, and compliance [35]. The placebo consisted of identical capsules, in which the bacterial cultures were replaced by maize starch. All four SYN‐53 variants were supplied by the sponsor, packaged indistinguishably, and differed neither in appearance nor taste.

At each intake cycle, participants took three capsules per day at mealtime for three consecutive days. The rescue medication was one tablet of cetirizine (10 mg), which could be taken within 24 h after an AEC session, if required. Intake of safety medication was monitored during safety calls.

### 2.4 Randomization and Masking

Subjects fulfilling all inclusion criteria were randomly assigned to either one of the three SYN‐53 variants or the placebo arm following a block randomization list. The randomization list was generated by an unmasked randomization administrator, who was independent of study conduct and data analysis. All participants, investigators, clinical monitors, project managers, and statisticians were masked to randomization. Rescue envelopes were in place for emergency unblinding.

### 2.5 Primary Endpoint and Further Outcome Parameters

During each 120‐min exposure, symptoms were assessed every 10 min using the Total Symptom Score (TSS, cf. Pfaar et al. [36]). TSS is composed of the Total Nose Symptom Score (TNSS, max. 12 points) and the Total Eye Symptom Score (TESS, max. 12 points), with a maximum possible score of 24 points. TNSS includes the symptoms of sneezing, itching, rhinorrhea, and nasal obstruction; TESS includes those of itching, foreign body feeling, tearing, and redness to the eyes (evaluated by the study nurse). The intensity of individual symptoms is scored from 0 to 3 (0 = no symptoms; 1 = mild symptoms; 2 = moderate symptoms; 3 = severe symptoms), summing up to a maximum of 12 points for each part score. The Maximum TSS (TSS_MAX_) is defined as the highest TSS recorded at any time point during allergen exposure. The primary endpoint was the change of TSS_MAX_ from baseline (E_1_) to post‐intervention (E_2_):

$${\text{∆TSS}}_{\mathrm{MAX}}\;\left(E_{2},E_{1}\right)={\mathrm{TSS}}_{\mathrm{MAX}}\;\left(E_{2}\right)-{\mathrm{TSS}}_{\mathrm{MAX}}\;\left(E_{1}\right)$$

Besides TESS and TNSS, the Total Bronchial Symptom Score (TBSS) and Total Other Symptom Score (TOSS) were assessed in an explorative manner. Tolerability and safety were evaluated based on the frequency and severity of adverse events (AEs) as well as on the need for emergency medication and intake discontinuations. This analysis was performed on the safety population containing those subjects that had received at least one dose of investigational product. AEs were recorded and evaluated using MedDRA (version 25.0) based on system organ classes (SOC) and preferred terms (PT).

### 2.6 Statistical Analysis

Based on the results of our previous study [34], a sample size of 34 subjects per group was calculated. A total of 40–42 participants per arm were included to increase the statistical power and to compensate for possible dropouts. A *p* value of *p* < 0.05 was considered statistically significant.

All statistical analyses were done by an independent biostatistician using SAS software version 9.4.

Efficacy evaluation was performed according to the intent‐to‐treat (ITT) principle. The ITT population included all randomized subjects. Missing values were replaced by missing at random (MAR) based multiple imputations, assuming monotonic missing values. Additionally, an analysis of completed cases (CC) was performed that included data of all participants completing exposures E_1_ and E_2_ in the AEC.

Analysis of covariance (ANCOVA) was used to evaluate the primary endpoint, that is, the reduction of TSS_MAX_ between E_2_ and E_1_ (∆TSS_MAX_), with ∆TSS_MAX_ as a factor and baseline value as a covariate, comparing the individual study arms with placebo. In advance, the Shapiro–Wilk test was applied to assess the distribution of the data. If normal distribution was not warranted, the ranked sums of the factor and covariate were used in preference to averaged data to perform a (non‐parametric) ANCOVA analysis. In addition, the primary endpoint was evaluated using the Wilcoxon rank sum test, comparing the individual study arms and placebo. For the analysis of other secondary/exploratory outcomes and demographic data, the Wilcoxon rank sum test (2 groups) or Kruskal–Wallis test (> 2 groups) were used.

## 3 Results

### 3.1 Study Population

**FIGURE 2:**
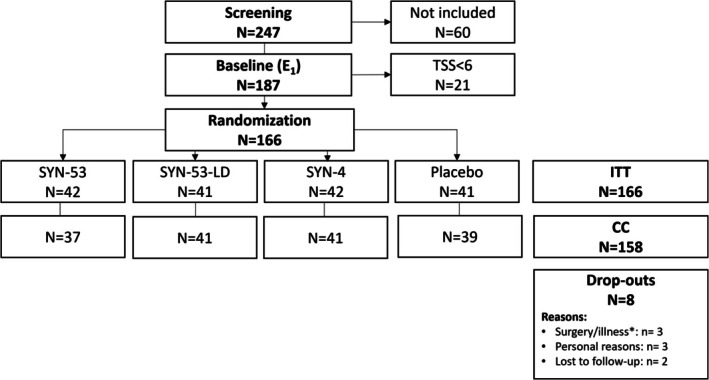
Overview of the study populations. ITT, Intent‐to‐treat population; CC, complete case population; TSS, Total Symptom Score. *Surgery/illness unrelated to study product.

A total of 247 subjects were screened at V_0_ . One hundred and eighty‐seven of the screened subjects had a relevant allergy history and a positive skin prick test and were admitted to baseline measurement (E_1_ ) in the AEC. One hundred sixty‐six of these subjects showed moderate‐to‐severe symptoms (TSS_MAX_ ≥ 6) and were randomized to receive either SYN‐53 (*n* = 42), SYN‐53‐LD (*n* = 41), SYN‐4 (*n* = 42), or a placebo (*n* = 41) (Figure 2). Subjects had a median age of 33.0 [27.0–40.0] (median [interquartile range]), a median BMI of 24.4 [22.3–26.8] kg/m^2^ and 68% were female. No statistical differences were observed across the study groups (cf. Table 1). The complete‐cases (CC) population included 158 subjects who finished all three intake cycles and the post‐intervention AEC exposure (E_2_ ). There were no major protocol violations. Eight participants dropped out of the study after randomization. All dropouts were unrelated to the study products (cf. Figure 2).

**TABLE 1:** Demographic data of the study groups. F = female, M = male, y = years, SPT = skin prick test, Ø mm = wheal diameter in millimeters, FEV1/FVC = ratio of the forced expiratory volume in the first 1 s in relation to the forced vital lung capacity. All values shown as median and interquartile range deviation. Kruskal–Wallis test.

	Placebo	SYN‐53	SYN‐53‐LD	SYN‐4	*p*
	41	42	41	42	
Gender					
F, *N* (%)	30 (73.2)	26 (61.9)	28 (68.3)	29 (69.0)	0.7423
M, *N* (%)	11 (26.8)	16 (38.1)	13 (31.7)	13 (31.0)	
Age [y]	33 (26–39)	36 (26–46)	31 (28–38)	31.5 (26–39)	0.5632
Body weight [kg]	70 (64–85)	75 (61–83)	70 (58–82)	72 (68–82)	0.5989
Height [cm]	171 (166–178)	170 (164–178)	170 (162–178)	175 (169–180)	0.1638
BMI [kg/cm^2^]	24.3 (22.6–26.7)	24.9 (22.7–26.8)	23.7 (21.7–27.0)	24.7 (22.3–26.8)	0.7577
% Smokers	19.5	9.5	9.8	11.9	0.4843
SPT [Ø mm]	10 (9–12)	9 (8–12)	10 (8–12)	10 (8–14)	0.3569
FEV1/FVC [%]	78 (73–81)	80 (76–82)	79 (75–82)	81 (73–84)	0.3662

### 3.2 Primary Endpoint

Baseline characteristics were similar between all study groups (*p* = 0.1380), as well as between the placebo, SYN‐53, SYN‐53‐LD, and SYN‐4 arms, respectively (cf. Table S2 for details).

**FIGURE 3:**
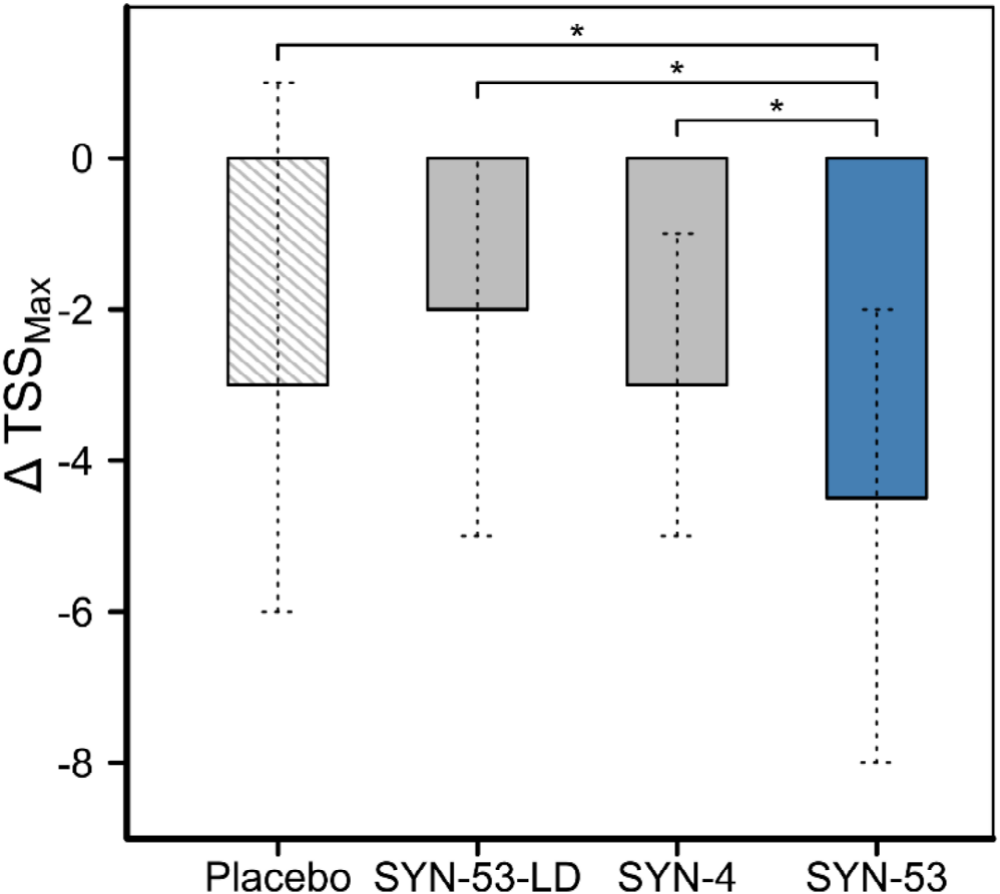
Changes of TSS_MAX_ from baseline upon intake of SYN‐53, SYN‐53‐LD, SYN‐4, or placebo of the intent‐to‐treat (ITT) population. Reduction of TSS_MAX_ from baseline depicted as ∆TSS_MAX_ of the ITT population for each study product. ∆TSS_MAX_ = TSS_MAX_ (E2)–TSS_MAX_ (E1). Data are shown as bar graph with mean and SE. *p* values were analyzed by nonparametric analysis of covariance (ANCOVA), on ranked sum effects of ∆TSS_MAX_ as factor and baseline values as covariate, comparing individual study arms with SYN‐53. Missing datapoints were replaced by MAR (missing at random) based multiple imputation. *n* (SYN‐53) = 42, *n* (SYN‐53‐LD) = 41, *n* (SYN‐4) = 42, and *n* (Placebo) = 41. *p > 0.05.

SYN‐53 was significantly superior in reducing TSS_MAX_ compared with its low dose variant SYN‐53‐LD (∆TSS_MAX_ [Mean ± SE]: −5.19 ± 0.80 vs. −2.27 ± 0.65; *p* = 0.0372), its low diversity variant SYN‐4 (−3.41 ± 0.52; *p* = 0.0482), and placebo (−2.82 ± 0.78; *p* = 0.0329) (cf. Figure 3 and Table S2). No significant differences to placebo were seen for either SYN‐53‐LD (*p* = 0.7377) or SYN‐4 (*p* = 0.5152). Similar results were obtained for the CC population (cf. Table S2 for details).

### 3.3 Further Outcome Parameters

Analysis of individual symptom scores (TESS, TNSS, TBSS, and TOSS) showed that both eye and nose symptoms contributed to the overall significant efficacy of SYN‐53, with particularly pronounced effects in eye symptoms (Table S3). The occurrence of adverse events (AEs) was monitored throughout the study. The safety population was identical to the CC population and included 158 subjects. All SYN‐53 variants were well tolerated, and adverse events were not different from placebo. A total of 15 AEs were reported (cf. Table 2), 11 of them graded as mild, three as moderate, and only one as severe. The severe AE occurred in the SYN‐53‐LD group and was a case of procedural headache during exposition in the AEC that recovered completely. None of the recorded events was reported to be related to the intake of the study product. No serious adverse events (SAE) were reported.

**TABLE 2:** Classification and incidence of adverse events.

		Placebo	SYN‐53	SYN‐53‐LD	SYN‐4	All
Injury, poisoning, and procedural complications	Procedural headache	1	—	1	1	3
	All	1	—	1	1	3
Infections and infestations	Nasopharyngitis	—	2	—	—	2
	Tonsilitis	—	1	—	—	1
	All	—	3	—	—	3
Gastrointestinal disorders	Dysphagia	—	—	2	—	2
	All	—	—	2	—	2
Skin and subcutaneous tissue disorders	Erythema	1	—	—	—	1
	Neurodermatitis	—	—	1	—	1
	All	1	—	1	—	2
General disorders and administration site conditions	Application site pain	—	1	—	—	1
	Fatigue	1	—	—	—	1
	All	1	1	—	—	2
Eye disorders	Dry eye	—	1	—	—	1
	All	—	1	—	—	1
Nervous system disorders	Headache	1	—	—	—	1
	All	1	—	—	—	1
Cardiac disorders	Palpitations	1	—	—	—	1
	All	1	—	—	—	1
All		5	5	4	1	15

## 4 Discussion

ARC is a chronic condition with increasing prevalence, and its symptoms severely impact the quality of life of millions of people worldwide. ARC imposes a significant economic burden on healthcare systems, warranting alternative approaches to ARC management [9, 10, 11, 12, 37, 38].

Following the latest evidence linking microbial composition and function to allergy susceptibility [22, 23, 25, 26, 27, 39, 40], the multi‐strain probiotic supplement SYN‐53 has been designed as a highly diverse composition of 53 distinct bacterial strains delivered at a concentration of 5 × 10^10^ CFU per capsule via a cyclic intake regime. SYN‐53 has recently been studied in the context of managing ARC in a randomized, placebo‐controlled clinical trial [34] and demonstrated significant alleviation of ARC and its symptoms compared with placebo, with symptom relief on par with conventional treatment options [41, 42, 43]. It was hypothesized that the effectiveness of SYN‐53 in the management of ARC is driven by its bacterial strain diversity and dosage. This follow‐up study aimed to reaffirm the efficacy and safety of SYN‐53, and to elucidate the impact of bacterial strain diversity and dosage by comparing it with a low dose variant (SYN‐53‐LD), a low diversity variant (SYN‐4), and a placebo. This is the second placebo‐controlled, randomized, double‐blind study to show that SYN‐53 significantly alleviates ARC and its symptoms. By adjusting the administration regime compared with [34], (1 week instead of two‐week cycles), symptom relief could be achieved even earlier, that is, in 3 weeks instead of 6, in this study. Despite adjustments in administration regimes, there is a remarkable consistency between the results of SYN‐53 in our study and those reported in [34], with both showing similar reductions in TSS_MAX_ (approximately 45% for SYN‐53 and 26% for the placebo) (Table S2). This consistency underscores the robustness of our findings. Notably, in both studies, the final allergen exposure occurred 1 week after the last administration of SYN‐53, demonstrating a sustained effect well beyond the 24‐h duration typical for conventional pharmacotherapies. Based on previous findings in [34], we hypothesize that the effectiveness of SYN‐53 increases progressively over time, resulting in prolonged therapeutic benefits extending over multiple weeks. Furthermore, no SAEs or study discontinuations related to the investigational product were reported in either of the two clinical studies with a total of over 250 subjects and multiple safety checkpoints. Much rather, all reported AEs in this study can be attributed to the natural course of ARC symptom development in the AEC. In neither study did adverse events differ between SYN‐53 and placebo. The ease of use and safety profile of SYN‐53 make it suitable for long‐term management of ARC.

Beyond reaffirming that SYN‐53 is a safe and effective novel strategy to manage the symptoms of ARC, this study demonstrates that the efficacy of this multi‐strain probiotic food supplement is intricately tied to bacterial strain diversity and dosage. Based on our findings, any modification to either of these two characteristics significantly impairs the effectiveness of SYN‐53 to a degree undistinguishable from placebo. The results of this study are of central importance to the further development of probiotic dietary supplements for ARC management, particularly given the high heterogeneity observed in clinical trials evaluating lower dose and lower diversity formulations [21, 22, 25, 26, 27]. In contrast to FMT, SYN‐53 can be reproducibly manufactured at scale and is not derived from human fecal matter that may contain food allergens or transmissible infectious agents. In addition, common side effects known for FMTs, such as abdominal distention, fatigue, constipation, chills, and diarrhea, have not been observed with SYN‐53.

Particular strengths of this study are its double‐blind design, its use of a state‐of‐the‐art AEC, and the robustness of its results. Unlike studies conducted outdoors, the AEC provides a standardized and controlled pollen challenge, enabling accurate and reproducible assessment of allergic symptoms, free from confounding environmental allergen exposure. The high quality and consistency of the study design also contributed to the remarkable alignment between the present findings and those of the preceding study by [34].

One limitation of the study is its restriction to adult subjects due to ethical considerations related to symptom induction in the AEC. However, the extensive safety documentation on the bacterial strains in SYN‐53 supports the extrapolation of tolerability to children, limiting the relevance of this exclusion. Moreover, this trial focused primarily on clinical symptoms of ARC. Future studies could explore the molecular mechanisms underlying the effects of SYN‐53.

The results of this study emphasize the potential of probiotics as a nutritional approach to the management of grass pollen allergy. Our findings highlight the essential role of the gut microbiome in maintaining immune homeostasis, paving the way for further research of well‐designed, high‐dosage, multi‐strain probiotic supplements in the management of further atopic diseases, such as asthma, dermatitis, or food allergies.

## Author Contributions

T.Z. and K.‐C.B. made substantial contributions to the conception and design of the study, and to the acquisition, analysis, and interpretation of data. Both authors were equally involved in drafting the manuscript. They agree to the version to be published and agree to be accountable for all aspects of the work, ensuring that any questions related to the accuracy or integrity of any part of the work are appropriately investigated and resolved. The sponsor participated in the study design. Data collation and statistical analysis were conducted by an independent clinical research organization (CRO).

## Conflicts of Interest

The European Centre for Allergy Research Foundation (ECARF) received reimbursement for participating as a study center. K.‐C.B. received honoraria for lectures from ALK, Allergopharma, Almirall, AstraZeneca, Bencard, Berlin‐Chemie, Chiesi, GSK, HAL, Mundipharma, Novartis, Sanofi, and Stallergenes during the last 5 years. T.Z. has received institutional funding for research and/or honoria for lectures and/or consulting from Amgen, AstraZeneca, AbbVie, ALK, Almirall, Astellas, Bayer Health Care, Bencard, Berlin Chemie, FAES, HAL, Henkel, Kryolan, Leti, L'Oreal, Meda, Menarini, Merck, MSD, Novartis, Pfizer, Sanofi, Stallergenes, Takeda, Teva, and UCB, Uriach; in addition, he is a member of ARIA/WHO, DGAKI, ECARF, GA2LEN, and WAO. The remaining authors declare no conflicts of interest.

## Supporting information


**Table S1:** Probiotic bacterial composition of study products SYN‐53, SYN‐53‐LD and SYN‐4. Species are labeled according to new systematic classification of Lactobacillus genera according to Zheng et al. 2020 (Zheng, Wittouck et al. 2020). Basonyms of the species are given in brackets. CFU, colony forming units. LD, low dose.
**Table S2:** Maximum Total Symptom Score (TSS_MAX_), ∆TSS_MAX_ of CC population. TSS‐values are shown as mean ± standard error and relate to the CC population. *p* values of ∆TSS_MAX_ are reported for the complete case (CC, § = Wilcoxon rank sum test comparing individual study arms with SYN‐53) population and the intent to treat (ITT, $ = Non‐parametric Analysis of covariance (ANCOVA) on ranked sum effects of ∆TSS_MAX_ as factor and baseline values as covariate comparing individual study arms with SYN‐53) population. Primary endpoint marked in bold. * = Relative reduction of TSS_MAX_ (% **∆**TSS_MAX_) is defined as percentage difference between E1 TSS_MAX_ and E2 TSS_MAX_. The relative reduction of TSS_MAX_ is not related to the statistical analysis and is provided for information purposes only.
**Table S3:** Change of individual symptom scores from baseline of complete case population (CC). Data represent mean with SE. All *p* values are given by ANCOVA based on ranks for the comparison of SYN‐53 symptom score vs. SYN‐53‐LD/SYN‐4/Placebo respectively and relate to CC population. TESS = total eye symptom score, TNSS = total nose symptom score, TBSS = total bronchial symptom score, TOSS = total other symptom score.

## Data Availability

The data that support the findings of this study are available from Synformulas. Restrictions apply to the availability of these data, which were used under license for this study. Data are available from https://synformulas.com/ with the permission of Synformulas.
