# Supplementary material for: Diversity and Dosage Determine the Efficacy of the Probiotic SYN‐53 in Allergic Rhinoconjunctivitis: A Randomized, Double‐Blind, Placebo‐Controlled Trial
Source: Allergy. 2025 Sep 10;80(12):3310–8. doi: 10.1111/all.70045 (PMC12666746; doi:10.1111/all.70045)
Supplement: Supplementary file 1 — Table S1: Probiotic bacterial composition of study products SYN‐53, SYN‐53‐LD and SYN‐4. Species are labeled according to new systematic classification of Lactobacillus genera according to Zheng et al. 2020 (Zheng, Wittouck et al. 2020). Basonyms of the species are given in brackets. CFU, colony forming units. LD, low dose. Table S2: Maximum Total Symptom Score (TSSMAX), ∆TSSMAX of CC population. TSS‐values are shown as mean ± standard error and relate to the CC population. p values of ∆TSSMAX are reported for the complete case (CC, § = Wilcoxon rank sum test comparing individual study arms with SYN‐53) population and the intent to treat (ITT, $ = Non‐parametric Analysis of covariance (ANCOVA) on ranked sum effects of ∆TSSMAX as factor and baseline values as covariate comparing individual study arms with SYN‐53) population. Primary endpoint marked in bold. * = Relative reduction of TSSMAX (% ∆TSSMAX) is defined as percentage difference between E1 TSSMAX and E2 TSSMAX. The relative reduction of TSSMAX is not related to the statistical analysis and is provided for information purposes only. Table S3: Change of individual symptom scores from baseline of complete case population (CC). Data represent mean with SE. All p values are given by ANCOVA based on ranks for the comparison of SYN‐53 symptom score vs. SYN‐53‐LD/SYN‐4/Placebo respectively and relate to CC population. TESS = total eye symptom score, TNSS = total nose symptom score, TBSS = total bronchial symptom score, TOSS = total other symptom score. [file ALL-80-3310-s001.docx]

**Supplementary material**

**Strain Diversity and Dosage Determine the Efficacy of the Probiotic SYN-53 in Allergic Rhinoconjunctivitis: Results from a Randomized, Placebo-Controlled Trial**

Karl-Christian Bergmann^1,2^ and Torsten Zuberbier^1,2*^

1. Institute of Allergology, Charité Universitätsmedizin, Berlin, Corporate Member of Freie Universität Berlin and Humboldt Universität zu Berlin, Berlin, Germany
2. Fraunhofer Institute for Translational Medicine and Pharmacology ITMP, Immunology and Allergology, Berlin, Germany

Supplementary Tables:

| **Study product variant** | **Bacterial strains** | **Dosage (CFU/capsule)** |
| --- | --- | --- |
| SYN-53 | Bifidobacterium bifidum BB02; Bifidobacterium bifidum SP9; Bifidobacterium adolescentis SP77; Lacticaseibacillus casei (Basonym: Lactobacillus casei) BGP93; Lacticaseibacillus casei (Basonym: Lactobacillus casei) LC10; Lacticaseibacillus casei (Basonym: Lactobacillus casei) LC11; Limosilactobacillus reuteri (Basonym: Lactobacillus reuteri) LR92; Bifidobacterium breve Bbr8; Bifidobacterium breve BL10; Lactobacillus crispatus SP28; Lacticaseibacillus rhamnosus (Basonym: Lactobacillus rhamnosus) SP1; Lacticaseibacillus rhamnosus (Basonym: Lactobacillus rhamnosus) LB21; Lacticaseibacillus rhamnosus (Basonym: Lactobacillus rhamnosus) IMC 501; Lacticaseibacillus rhamnosus (Basonym: Lactobacillus rhamnosus) LR1; Lacticaseibacillus rhamnosus (Basonym: Lactobacillus rhamnosus) LRH01; Lacticaseibacillus rhamnosus (Basonym: Lactobacillus rhamnosus) LRH05; Lacticaseibacillus rhamnosus (Basonym: Lactobacillus rhamnosus) LRH14; Lacticaseibacillus rhamnosus (Basonym: Lactobacillus rhamnosus) LRH58; Bifidobacterium longum ssp. longum SP54; Limosilactobacillus fermentum (Basonym: Lactobacillus fermentum) LF2; Limosilactobacillus fermentum (Basonym: Lactobacillus fermentum) CS57; Ligilactobacillus salivarius (Basonym: Lactobacillus salivarius) SP2; Bifidobacterium animalis ssp.lactis BLC1; Bifidobacterium animalis ssp.lactis Bi1; Lactococcus lactis ssp. lactis SP38; Lactococcus lactis ssp. lactis SP47; Lactococcus lactis ssp. lactis SD13; Lactobacillus lactis ssp. lactis SL97; Lactobacilus helveticus SP27; Lactobacillus helveticus LH102; Streptococcus thermophilus SP4; Streptococcus thermophilus Z57; Streptococcus thermophilus ST628; Lactobacillus acidophilus LA3; Lactobacillus acidophilus LA1; Lentilactobacillus buchneri subsp. buchneri (Basonym: Lactobacillus buchneri) LBC01; Lactobacillus delbrueckii ssp. bulgaricus LB2; Lactobacillus delbrueckii ssp. bulgaricus LB284; Lactiplantibacillus plantarum subsp. plantarum (Basonym: Lactobacillus plantarum) 14D; Lactiplantibacillus plantarum subsp. plantarum (Basonym: Lactobacillus plantarum) LB931; Lactiplantibacillus plantarum subsp. plantarum (Basonym: Lactobacillus plantarum) BG112; Lactiplantibacillus plantarum subsp. plantarum (Basonym: Lactobacillus plantarum) LP48; Pediococcus acidilactici PA09; Lacticaseibacillus paracasei subsp. paracasei (Basonym: Lactobacillus paracasei) IMC 502; Lacticaseibacillus paracasei subsp. paracasei (Basonym: Lactobacillus paracasei) 101/37; Lacticaseibacillus paracasei subsp. paracasei (Basonym: Lactobacillus paracasei) BGP1; Lacticaseibacillus paracasei subsp. paracasei (Basonym: Lactobacillus paracasei) BGP2; Lacticaseibacillus paracasei subsp. paracasei (Basonym: Lactobacillus paracasei) LPC43; Pediococcus pentosaceus PP02; Levilactobacillus brevis (Basonym: Lactobacillus brevis) SP48; Latilactobacillus sakei subsp. sakei (Basonym: Lactobacillus sakei) LSK04; Latilactobacillus sakei subsp. sakei (Basonym:Lactobacillus sakei) LSK14; Lactobacillus delbrueckii ssp. lactis (Basonym: Lactobacillus lactis) LL82 | 5 x 10^10^ |
| SYN-53-LD | Bifidobacterium bifidum BB02; Bifidobacterium bifidum SP9; Bifidobacterium adolescentis SP77; Lacticaseibacillus casei (Basonym: Lactobacillus casei) BGP93; Lacticaseibacillus casei (Basonym: Lactobacillus casei) LC10; Lacticaseibacillus casei (Basonym: Lactobacillus casei) LC11; Limosilactobacillus reuteri (Basonym: Lactobacillus reuteri) LR92; Bifidobacterium breve Bbr8; Bifidobacterium breve BL10; Lactobacillus crispatus SP28; Lacticaseibacillus rhamnosus (Basonym: Lactobacillus rhamnosus) SP1; Lacticaseibacillus rhamnosus (Basonym: Lactobacillus rhamnosus) LB21; Lacticaseibacillus rhamnosus (Basonym: Lactobacillus rhamnosus) IMC 501; Lacticaseibacillus rhamnosus (Basonym: Lactobacillus rhamnosus) LR1; Lacticaseibacillus rhamnosus (Basonym: Lactobacillus rhamnosus) LRH01; Lacticaseibacillus rhamnosus (Basonym: Lactobacillus rhamnosus) LRH05; Lacticaseibacillus rhamnosus (Basonym: Lactobacillus rhamnosus) LRH14; Lacticaseibacillus rhamnosus (Basonym: Lactobacillus rhamnosus) LRH58; Bifidobacterium longum ssp. longum SP54; Limosilactobacillus fermentum (Basonym: Lactobacillus fermentum) LF2; Limosilactobacillus fermentum (Basonym: Lactobacillus fermentum) CS57; Ligilactobacillus salivarius (Basonym: Lactobacillus salivarius) SP2; Bifidobacterium animalis ssp.lactis BLC1; Bifidobacterium animalis ssp.lactis Bi1; Lactococcus lactis ssp. lactis SP38; Lactococcus lactis ssp. lactis SP47; Lactococcus lactis ssp. lactis SD13; Lactobacillus lactis ssp. lactis SL97; Lactobacilus helveticus SP27; Lactobacillus helveticus LH102; Streptococcus thermophilus SP4; Streptococcus thermophilus Z57; Streptococcus thermophilus ST628; Lactobacillus acidophilus LA3; Lactobacillus acidophilus LA1; Lentilactobacillus buchneri subsp. buchneri (Basonym: Lactobacillus buchneri) LBC01; Lactobacillus delbrueckii ssp. bulgaricus LB2; Lactobacillus delbrueckii ssp. bulgaricus LB284; Lactiplantibacillus plantarum subsp. plantarum (Basonym: Lactobacillus plantarum) 14D; Lactiplantibacillus plantarum subsp. plantarum (Basonym: Lactobacillus plantarum) LB931; Lactiplantibacillus plantarum subsp. plantarum (Basonym: Lactobacillus plantarum) BG112; Lactiplantibacillus plantarum subsp. plantarum (Basonym: Lactobacillus plantarum) LP48; Pediococcus acidilactici PA09; Lacticaseibacillus paracasei subsp. paracasei (Basonym: Lactobacillus paracasei) IMC 502; Lacticaseibacillus paracasei subsp. paracasei (Basonym: Lactobacillus paracasei) 101/37; Lacticaseibacillus paracasei subsp. paracasei (Basonym: Lactobacillus paracasei) BGP1; Lacticaseibacillus paracasei subsp. paracasei (Basonym: Lactobacillus paracasei) BGP2; Lacticaseibacillus paracasei subsp. paracasei (Basonym: Lactobacillus paracasei) LPC43; Pediococcus pentosaceus PP02; Levilactobacillus brevis (Basonym: Lactobacillus brevis) SP48; Latilactobacillus sakei subsp. sakei (Basonym:Lactobacillus sakei) LSK04; Latilactobacillus sakei subsp. sakei (Basonym:Lactobacillus sakei) LSK14; Lactobacillus delbrueckii ssp. lactis (Basonym: Lactobacillus lactis) LL82 | 6 x 10^9^ |
| SYN-4 | Bifidobacterium bifidum BB02; Lacticaseibacillus rhamnosus (Basonym: Lactobacillus rhamnosus) SP1; Bifidobacterium animalis ssp.lactis Bi1; Lacticaseibacillus paracasei subsp. paracasei (Basonym: Lactobacillus paracasei) 101/37 | 4 x 10^10^ |

**Table S1:** Probiotic bacterial composition of study products SYN-53, SYN-53-LD and SYN-4. Species are labeled according to new systematic classification of Lactobacillus genera according to Zheng et al., 2020 (Zheng, Wittouck et al. 2020). Basonyms of the species are given in brackets. CFU, colony forming units. LD, low dose.

| **Group** | **N**  **_ITT/CC_** | **E_1_ TSS_MAX_** | ***p***^§^ | **E_2_ TSS_MAX_** | **%**  **∆TSS_MAX*_** | **∆TSS_MAX_** | ***P***^§^  ^vs. SYN-53^ | ***p***^$^  ^vs. SYN-53^ |
| --- | --- | --- | --- | --- | --- | --- | --- | --- |
| **SYN-53** | 42/37 | 11.84  ± 0.79 | - | 6.65  ± 0.64 | 44% | - 5.19  ± 0.80 | - | **-** |
| **SYN-53-LD** | 41 / 41 | 9.95  ± 0.52 | 0.1051 | 7.68  ± 0.64 | 23% | - 2.27  ± 0.65 | 0.0097 | **0.0372** |
| **SYN-4** | 42 / 41 | 11.46  ± 0.59 | 0.9760 | 8.05  ± 0.64 | 30% | - 3.41  ± 0.52 | 0.0992 | **0.0482** |
| **Placebo** | 41 / 39 | 10.74  ± 0.53 | 0.5109 | 7.92  ± 0.62 | 26% | - 2.82  ± 0.78 | 0.0335 | **0.0329** |

***Table S2.*** *Maximum Total Symptom Score (TSS_MAX_), ∆TSS_MAX_ of CC-population. TSS-values are shown as mean ± standard error and relate to the CC population.* *p-values of ∆TSS_MAX_ are reported for the complete case (CC, § = Wilcoxon rank sum test comparing individual study arms with SYN-53) population and the intent to treat (ITT, $ = Non-parametric Analysis of co-variance (ANCOVA) on ranked sum effects of ∆TSS_MAX_ as factor and baseline values as covariate comparing individual study arms with SYN-53) population. Primary endpoint marked in bold. * = Relative reduction of TSS_MAX_ (%* **∆***TSS_MAX_) is defined as percentage difference between E1 TSS_MAX_ and E2 TSS_MAX_. The relative reduction of TSS_MAX_ is not related to the statistical analysis and is provided for information purposes only.*

| **Group** | **N CC** | **Symptom**  **score** | **Mean ± SE** | **p-value**  ^vs. SYN-53^ |
| --- | --- | --- | --- | --- |
| **SYN-53** | 37 | **∆**TESS_MAX_ | -2.89  ± 0.44 | - |
|  |  | **∆**TNSS_MAX_ | -2.19  ± 0.46 | - |
|  |  | **∆**TBSS_MAX_ | -1.59  ± 0.43 | - |
|  |  | **∆**TOSS_MAX_ | -0.95  ± 0.17 | - |
| **SYN-53-LD** | 41 | **∆**TESS_MAX_ | -1.12  ± 0.43 | 0.0374 |
|  |  | **∆**TNSS_MAX_ | -1.27  ± 0.43 | 0.3933 |
|  |  | **∆**TBSS_MAX_ | -1.12  ± 0.34 | 0.0499 |
|  |  | **∆**TOSS_MAX_ | -0.63  ± 0.18 | 0.2654 |
| **SYN-4** | 41 | **∆**TESS_MAX_ | -1.49  ± 0.35 | 0.0229 |
|  |  | **∆**TNSS_MAX_ | -2.02  ± 0.35 | 0.6878 |
|  |  | **∆**TBSS_MAX_ | -1.49  ± 0.38 | 0.7544 |
|  |  | **∆**TOSS_MAX_ | -1.02  ± 0.20 | 0.5659 |
| **PLACEBO** | 39 | **∆**TESS_MAX_ | -1.59  ± 0.43 | 0.2209 |
|  |  | **∆**TNSS_MAX_ | -1.10  ± 0.46 | 0.1162 |
|  |  | **∆**TBSS_MAX_ | -0.67  ± 0.27 | 0.7619 |
|  |  | **∆**TOSS_MAX_ | -0.77  ± 0.23 | 0.2392 |

***Table S3:*** *Change of individual symptom scores from baseline of complete case population (CC). Data represent mean with SE. All P-values are given by ANCOVA based on ranks for the comparison of SYN-53 symptom score vs. SYN-53-LD/SYN-4/Placebo respectively and relate to CC-population. TESS = total eye symptom score, TNSS = total nose symptom score, TBSS = total bronchial symptom score, TOSS = total other symptom score.*

**References**

Zheng, J., S. Wittouck, E. Salvetti, C. M. A. P. Franz, H. M. B. Harris, P. Mattarelli, P. W. O’Toole, B. Pot, P. Vandamme, J. Walter, K. Watanabe, S. Wuyts, G. E. Felis, M. G. Gänzle and S. Lebeer (2020). "A taxonomic note on the genus Lactobacillus: Description of 23 novel genera, emended description of the genus Lactobacillus Beijerinck 1901, and union of Lactobacillaceae and Leuconostocaceae." International Journal of Systematic and Evolutionary Microbiology **70**(4): 2782-2858.
